# Supplementary material for: Real-time biodiversity analysis using deep-learning algorithms on mobile robotic platforms
Source: PeerJ Comput Sci. 2023 Aug 25;9:e1502. doi: 10.7717/peerj-cs.1502 (PMC10495972; doi:10.7717/peerj-cs.1502)
Supplement: Supplemental Information 1 [file peerj-cs-09-1502-s001.pdf]

Supplementary Material

The multimedia presentation of the work is available to visualise the results of the proposed algorithm on the validation dataset as a video. The code repository and the utilised dataset is also made open-source to make the findings available to fellow ecologists and researchers for further study and extensive research. The comparison of the ground truth labels alongside the predicted results from multiple algorithms proposed in this study is illustrated in Fig. S1 for further reference.

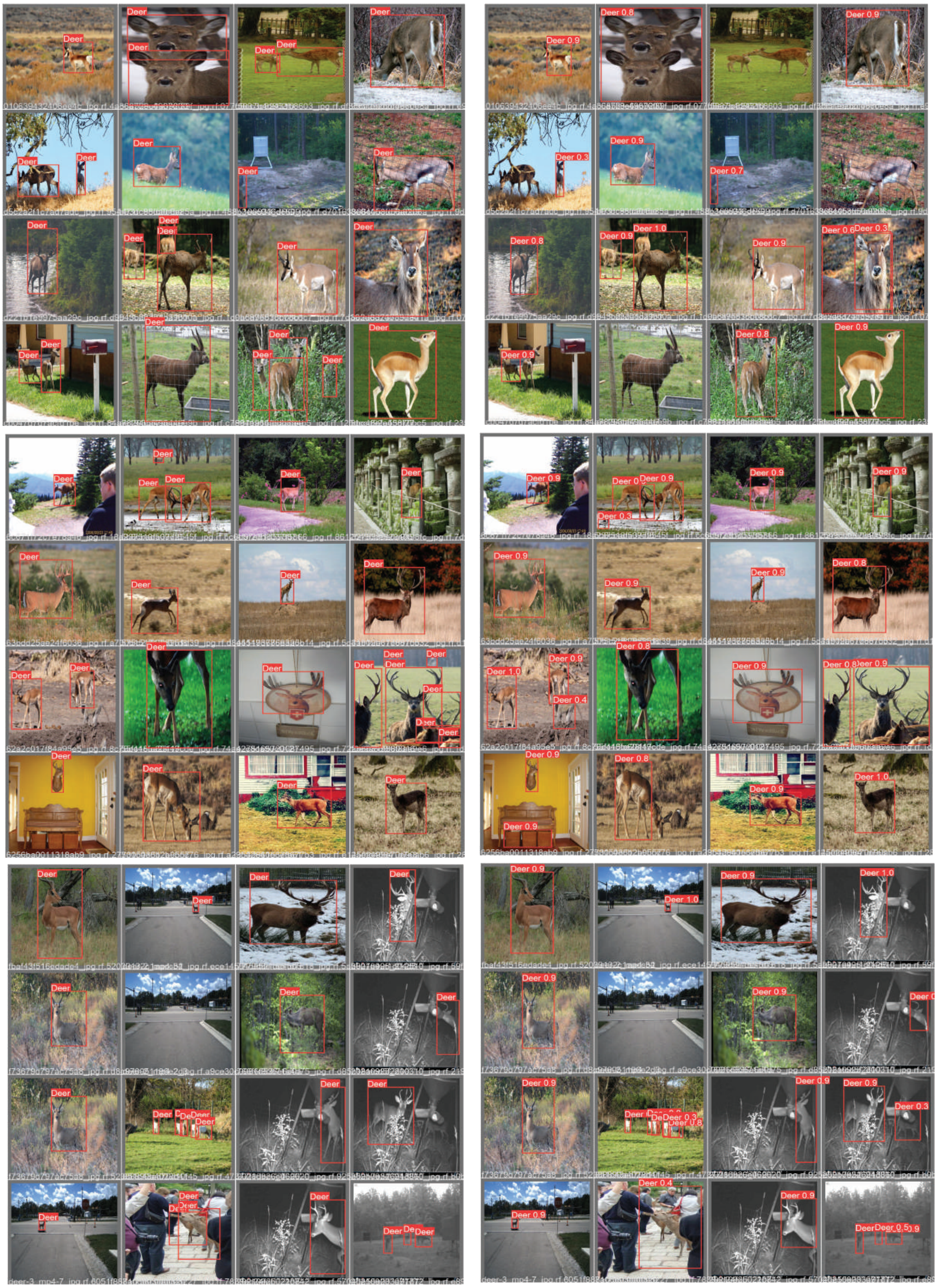

Figure S1: Comparison of the predicted results from different algorithms alongside the ground-truth labels.
